# Neuroecotoxicology: Effects of environmental heavy metal exposure on the brain of African giant rats and the contribution of vanadium to the neuropathology

**DOI:** 10.1016/j.ibneur.2022.08.008

**Published:** 2022-08-30

**Authors:** Ifukibot Levi Usende, James Olukayode Olopade, Idris Ayodeji Azeez, Anna Andrioli, Molakun O. Bankole, Funmilayo E. Olopade, Allam A. Nafady, Marina Bentivoglio

**Affiliations:** a Department of Veterinary Anatomy, University of Abuja, Nigeria; b Department of Veterinary Anatomy, University of Ibadan, Nigeria; c Department of Veterinary Anatomy, University of Jos, Nigeria; d Department of Neurological and Movement Sciences, University of Verona, Italy; e Department of Human Anatomy, College of Medicine, University of Ibadan, Nigeria; f Department of Veterinary Pathology, Electron Microscope Unit, Assuit University, Egypt

**Keywords:** Neuroecotoxicology, Nigeria, Agro-ecological zones, Heavy metals, Neuronal cell degeneration, African giant rats

## Abstract

Increased exploitation of minerals has led to pollution of confined environments as documented in Nigeria Niger Delta. Information on the effects on brain of such exposure is limited. Due to its exploratory activities, the African giant rat (*Cricetomys gambianus*) (AGR) provides a unique model for neuroecotoxicological research to determine levels of animal and human exposure to different pollutants. This study aims to unravel neuropathological features of AGR sampled from three agro-ecological zones of Nigeria. Fifteen AGR were sampled according to previously determined data on heavy metal exposure: high vanadium, high lead, and low metals. Eighteen AGR were collected from low metal zone and divided into two groups. Control group received vehicle while SMV exposed group received 3 mg/kg sodium metavanadate (SMV) intraperitoneally for 14days. Brain immunohistochemical analyses were conducted, and ultrastructural changes were studied in experimentally exposed group. Results showed significant loss of tyrosin hydroxylase, parvalbumin, orexin-A and melanin concentration hormone containing neuronal populations in brains obtained from high vanadium and high lead zones and in experimentally intoxicated SMV groups. Similarly, significant decrease numbers of dendritic arborations; extracellular matrix density, perineuronal nets; astrocytes and microglia activations are documented in same groups. Ultrastructural studies revealed mass denudation, cilia loss, disintegration of ependymal layer and intense destructions of myelin sheaths in SMV exposed group. These are the first “neuroecotoxicological” findings in distinct neuronal cells. The implications of these findings are highly relevant for human population living in these areas, not only in Nigeria but also in similarly polluted areas elsewhere in the world.

## 1 Introduction

Increased exploitation of minerals has led to pollution of confined environments (Igado et al., 2008, Olopade et al., 2005), as documented in the Niger Delta in Nigeria (Usende et al., 2016, Usende et al., 2017, Usende et al., 2020, Usende et al., 2022). Air pollution which is a complex mixture of gases, metals, indoor and outdoor organic compounds, endotoxins and particulate matter is associated with adverse health effects (Calderon-Garciduenas et al., 2001, Calderon-Garciduenas et al., 2002). In Mexico City for example, Calderon-Garciduenas et al. (2002) have reported chronic respiratory tract inflammation, breakdown of the nasal and olfactory barriers as well as the blood brain barrier (BBB) in canine species exposed naturally to polluted environment leading to pathologies. However, despite the increasing industrial exploitation of minerals and its consequent environmental pollution in Nigeria (Olopade et al., 2005, Usende et al., 2016, Usende et al., 2017), there has been poor monitoring of the adverse effects of such exposure, especially concerning neuropathological effects. Previous studies have indicated that domestic and wildlife animals living in polluted environments represents a very important biological source of data for assessing risks to human health (Schilderman et al., 1997; Calderon-Garciduenas et al., 2002, Calderon-Garciduenas et al., 2003; Usende et al., 2017; Jubril et al., 2019) including brain health.

The African giant rat (*Cricetomys gambianus,* Waterhouse, 1840; family *Muridae*), ubiquitous in Central and West African countries, including Nigeria, is a pouched rodent (Ibe et al., 2014). Due to its nocturnal nature and exploratory activities, this animal can provide a unique model for neuroecotoxicological research to determine the levels of animal and human exposure to environmental pollutants. Specifically, the African giant rat, represent an important source of information that could be applied to the understanding of the neuropathologies potentially affecting about more than thirty million people (both adult and children) (Stakeholder Democracy Network (SDN), 2020) currently living in the various polluted cities of Niger Delta, Nigeria. Indeed, we have recently shown, using atomic absorption spectrophotometry, different levels of heavy metals concentration in tissues, including the brain, of African giant rats living in regions of Nigeria characterized by different industrial activities and consequent heavy metal pollution (Usende et al., 2017). In particular, a three-fold increase in vanadium concentration was found in brain tissue of African giant rats sampled in the mangrove/fresh water swamp agro-ecological zone (Niger delta region) and a two-fold increase in lead concentration of brain tissues of African giant rats sampled in the woodland/tall grass savanna agro-ecological zone (Northern region) compared to exposure to the relatively low pollution of the rain forest agro-ecological zone (Western region) of Nigeria (Usende et al., 2017). The aim of this study therefore is to investigate the neuropathological features of African giant rats sampled in the field from three agro-ecological zones of Nigeria and also to ascertain the contribution of vanadium to such neuropathology. The translational implications of such findings are highly relevant for the human population living in the same areas, not only in Nigeria but also in similarly polluted areas elsewhere in the world.

## 2 Materials and methods

### 2.1 Study areas

The sampling points were as described by Usende et al. (2017) and correspond to three out of six agro-ecological zones of Nigeria with different explorative activities. The design was aimed to reflect neuropathologies due to two major environmental heavy metal contaminants (vanadium and lead) of these agro-ecological zones. A brief description of the study points are as follows:

#### 2.1.1 Mangrove/fresh water swamp agro-ecological (High vanadium) zone (n = 5)

Ethiop served as sampling site for this zone. The city is located in the coastal zone of the Nigeria Niger Delta and several kilometres away from the shores of the Atlantic Ocean. The city is hub of petroleum refining activities and therefore experience major oil spillage and gas flaring (Adati, 2012, Usende et al., 2017).

#### 2.1.2 Woodland and tall grass savanna agro-ecological (High lead) zone (n = 5)

Gwagwalada served as the sampling site for this zone. Located in Federal Capital Territory of Nigeria, the city is influential and important in various socio-economic activities including excavating and illegal gold mining and environmental pollution (Usende et al., 2017).

#### 2.1.3 Rain forest agro-ecological (Low metal) zone (n = 23)

Ibadan served as the sampling site for this zone. The city is located in the South western part of Nigeria. The major industries activities common in Ibadan include food and beverage processing, auto repair workshops, and organic and agro-allied chemicals manufacturing, pharmaceuticals among others (Osibanjo et al., 2011).

### 2.2 Neuroecotoxicological study

Once captured, the AGR were deeply anaesthetized with ketamine and xylazine (90/10 mg/kg, i.p). They were transported in ventilated cages to Neuroscience laboratory at the University of Ibadan in the case of 1–2 h journey (from the Rain forest) and perfused transcardially (n = 5) as described by Usende et al., 2016, Usende et al., 2020. Otherwise, a perfusion apparatus was set up in the field for animals captured from the Mangrove/Fresh water swamp agro-ecological (high vanadium, n = 5) and Woodland and tall grass savanna agro-ecological (high lead, n = 5).

### 2.3 Experimental Sodium metavanadate treatment

We have determined in pilot experiments that African giant rats are difficult to breed in animal facilities, in which, however, they easily survive. African giant rats (n = 18) from the Rain Forest agro-ecological zone (low metals level) were used to investigate brain damage caused by experimental vanadium exposure. The animals were captured as above and transported in ventilated cages to the Neuroscience laboratory at the University of Ibadan. The animals were acclimated for two weeks, and randomly divided into two groups (*n* = 9 per group).

Group 1 served as the SMV treated group and received intraperitoneal (i.p) injection of SMV at 3 mg/kg body weight (Usende et al., 2016, Usende et al., 2018a, Usende et al., 2018b, Usende et al., 2020, Usende et al., 2022) for 14 consecutive days. The dose of 3 mg/kg body weight used in this study has been shown not to record mortality but induced hypomyelination and neurobehavioural deficits in Sprague Dawley rats; and oxidative stress, hepatorenal and cytogenotoxicity in AGR (Garcia et al., 2004, Usende et al., 2016, Usende et al., 2018a, Usende et al., 2018b, Usende et al., 2020, Usende et al., 2022). Group 2 served as control and received sterile injection water i.p for 14 days. All animal experiments were carried out under ethical approval of the University of Ibadan Animal Care and Use Research Ethics Committee Review (UI-ACUREC/18/0059) and in accordance with ethical standard of the National Institute of Health Guide for the Care and Use of Laboratory Animals (NIH Publications No. 8023) and the European Communities Council Directive of November 24, 1986 (86/609/EEC). Twenty hours after the last treatment, all animals were perfused using 4 % phosphate-buffered paraformaldehyde. The protocols used for animal perfusion was as described by Usende et al., 2016, Usende et al., 2020. For the experimentally exposed protocol, five (5) AGR from SMV treated and five (5) from their control match groups were used for immunohistochemical studies while four (4) from each group were used for electron microscopy.

#### 2.3.1 Immunohistochemistry

The brains were carefully dissected out immediately after perfusion, post-fixed in same 4 % phosphate-buffered paraformaldehyde for 24 h, and then stored in the lab at 4 °C in 0.1 % sodium azide in 0.01 M phosphate buffered saline (PBS) until time of processing.

At the time of processing, the brains were cryoprotected in 30 % sucrose and stored at 4° C for 3 days. Cryoprotected brains were then cut on a freezing microtome into serial coronal sections at 40 µm thickness. All sections from the olfactory bulb through the hypothalamus were collected in series of every fifth section. Sections were then processed free-floating for immunohistochemical procedures.

The following primary antibodies were used: rabbit anti PV polyclonal antibodies (1:1000; Santa Cruz Biotechnology, Santa Cruz, CA, USA); mouse anti-TH antibodies (1:1000; Santa Cruz Biotechnology); rabbit anti- OX-A polyclonal antibodies (1:1000; Santa Cruz Biotechnology, Santa Cruz, CA, USA); and rabbit anti-MCH antibodies (1:1000; Phoenix Pharmaceutical, Belmont, CA, USA). In addition, rabbit anti-IBA1 antibodies (1:500; Chemicon, Temecula, CA, USA) were used for the study of microglia, and rabbit anti-GFAP antibodies (1:500; Chemicon) were used to study astrocytes. Biotinylated horse anti-mouse and horse anti-rabbit IgGs (1:200; Vector Laboratories, Burlingame, CA, USA) were used as secondary antibodies. Immunoreactivity was visualized using the Vectastain ABC Elite kit (Vector Laboratories) and 3,30- diaminobenzidine as chromogen. Sections were mounted on gelatinized slides, dehydrated in graded ethanol, cleared in xylene, coverslipped and allowed to dry before examination (Usende et al., 2013).

In addition, brain sections were processed for double immunofluorescence to reveal simultaneously parvalbumin neurons and perineuronal nets (PNNs) and extracellular matrix (ECM) expression. These sections were pre-incubated for 1 h in a blocking solution of 2% normal horse serum (NHS) with 0.2 % Triton X-100 in PBS. Sections were subsequently incubated overnight at 4 °C in rabbit anti PV polyclonal antibodies (1:1000; Santa Cruz Biotechnology, Santa Cruz, CA, USA) and biotinylated WFA (Sigma Aldrich; 1:200), which was used to visualize ECM. Primary antibodies were diluted in PBS containing 1% NHS and 0.2 % Triton X-100. The slides were later incubated for 2 h in Avidin-488 and DaRb-568 (all from Invitrogen, Carlsbad, CA, USA) secondary antibodies diluted 1:1000 in 1 % NHS. Sections were mounted on gelatinized slides and coverslipped with 0.1 % paraphenylenediamine in a glycerol-based medium (90 % glycerol and 10 % PBS). For quality control, additional sections were used, omitting each of the primary antibodies and no specific immunostaining was observed in these sections.

#### 2.3.2 Quantitative analyses

##### 2.3.2.1 Cell counts

To quantity cell population, quantitative analyses were done blinded to experimental conditions, with an Olympus BX51 microscope connected to a digital camera (JVC CCD KY-F58) and equipped with Quantitative Neuron Analysis software, Neurolucida® 360 (Mbf, Bioscience), using all brains from high vanadium, high lead and low metals as well as brains from 3 mg/kg SMV treated and control groups AGR. Unbiased counts of labeled neurons (TH immunoreactive neurons in the SNc; PV immunoreactive neurons in the prefrontal cortex, hippocampus and reticular thalamic area; OX-A and MCH-immunoreactive neurons in the LH) were performed in sections regularly spaced throughout the rostrocaudal extent of these regions (at 240-µm intervals). Cell counting were performed (Gerashchenko et al., 2001, Gaykema and Goehler, 2009) using optical fractionator method (West, 1993).

##### 2.3.2.2 Analysis of dendritic architecture OX-A immunopositive neurons

Changes of dendritic arbors of OX-A neurons in brains from high vanadium, high lead and low metals as well as brains from 3 mg/kg SMV intoxicated and control groups AGR were analyzed quantitatively with the semi-automated Sholl analysis using the software Image J (NIH, Bethesda, MD, USA), as described by Ferreira et al. (2014) and Palomba et al. (2015). Briefly, individual OX-A neurons selected were analyzed by setting a center point in the soma. From this point (in the soma), concentric circles (with increasing radius) of 1 µm and at a constant interval were automatically drawn by the software, and the number of dendritic intersections within each circle was counted. The mean intersections (which indicates the average number of dendritic intersections within concentric circles), the critical value (i.e., the mean distance from the soma where the highest number of intersections is found), the ramification index (i.e., the average number of dendrites originating in proximity of the soma), the critical radius (i.e., the lowest radius formed by dendrites clustering together before a new phase) as well as the intersecting and ending radii were also evaluated.

#### 2.3.3 Analysis of integrated density of WFA around soma of PV immunopositive neurons

Immunofluorescence sections were viewed with the confocal microscope Leica SP5 (Leica, Mannheim, Germany) using X40 (zoom factor 1.9) or 63X (zoom factor 1.8) oil-immersion objective (numerical aperture 1.25). Serial Z planes (0.70 µm) images were captured with the LAS software, and collapsed into a single maximum projection image to which colours were assigned. Acquired images were minimally and equally modified with the Imaris software 7.4 (Bitplane, Zurich, Switzerland) for contrast and brightness.

To evaluate the PNN degradation around PV immunopositive neurons of the prefrontal cortex, we quantified the immunofluorescence data of WFA intensity using the software Image J (NIH, Bethesda, MD, USA). In brief, we averaged the mean fluorescence intensities around the soma and dendrites of each neuron. For each of 5 adjacent brain sections per animal per group, 4 individual neurons were used by an investigator blinded to the experimental protocol. Individual PNNs was used to overcome the potential discrepancy due to different density of PNNs and neurons in similar dimension areas (Bhanu et al., 2018). Subsequently, the mean intensities from all 4 neurons from 5 brain slices from 5 AGR per group were averaged to obtain mean ± SEM and the numbers of analyzed brain slices were considered as n for statistical analysis.

### 2.4 Scanning electron microscopy

Samples for SEM were processed according to protocol described by Hoeflich et al. (2002) and Usende et al., 2020, Usende et al., 2022 with little modifications. Briefly, samples were carefully washed to remove debris from surface and were transferred to glass bottles, rinsed in 0.1 M cacodylate buffer and then post-fixed in 1 % osmium tetraoxide for 4 h. Brain samples were then dehydrated through graded alcohol, cleared in acetone, and freeze dried in amyl acetate to substitute for the water in the tissues. The samples were then dried in a thermed 5001 electronic (Jurgens) critical point drier using carbon-dioxide as transitional fluid, mounted with carbon conductive cement (CCC) adhesive, gold coated in a JEOL (JFC-1100E) ion sputtering device and viewed using JEOL (JSM-5400LV) scanning electron microscopy. Interest was focused on the floor of the lateral ventricles.

### 2.5 Transmission electron microscopy

Protocol for TEM was as described by Hoeflich et al. (2002) and modified by Usende et al., 2020, Usende et al., 2022. The 1–2 mm slices of brain samples were transferred to glass bottles, rinsed in 0.1 M cacodylate buffer and then post-fixed in 1 % osmium tetraoxide for 4 h. Brain samples were then dehydrated through increasing grades of alcohol, embedded in Epon and semi-thin sections were obtained with LKB (8800 ultrotome) microtome. The semi-thin sections were stained with toluidin blue for examination of region of interest (ROI) using Olympus CX31 light microscope. Ultrathin section (60–80 nm) were then obtained based on the ROI with Reichert- ultracut S (Leica) microtome, placed on copper grids, and stained with uranyl acetate and lead citrate. Stained sections were descriptively analyzed with a JEOL (JEM-100CXII) transmission electron microscope and photographs were obtained with a CCD digital camera (model XR-41M).

### 2.6 Statistical evaluation

Statistics were performed using GraphPad Prism version 7.0 software and a *P* value of <0.05 was accepted as statistically significant. All numerical data generated were evaluated for statistical significance using one-way ANOVA with Tukey’s multiple post-test comparison for the three zones (high vanadium, high lead and low metals) under study. SMV treated group was compared to control group by unpaired T-test. Also, natural vanadium exposed (high vanadium zone) was compared to experimental exposed SMV group using unpaired T-test.

## 3 Results

### 3.1 Clinical and gross pathological findings

All African giant rats from the three zones used in this study were apparently healthy without any physical injury. The body weight range of the AGR was between 700 and 840 g.

### 3.2 Neuroecotoxicological findings in distinct neuronal cells of AGR sampled from their natural environment

#### 3.2.1 Substantia nigra pars compacta (SNC) dopaminergic neuron

**Fig. 1:**
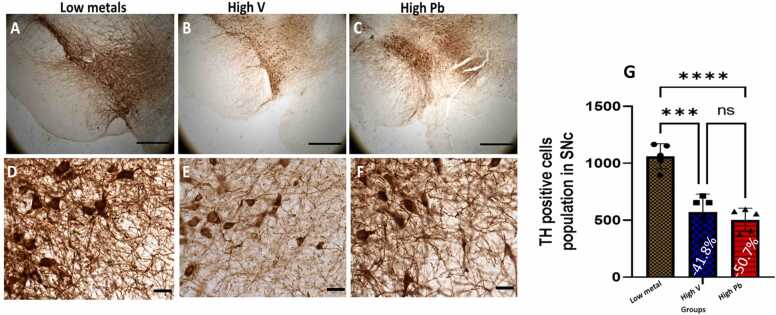
Tyrosine hydroxylase (TH) immunoreactivity of dopaminergic neurons in the substantia nigra pars compacta (SNc) of AGR sampled from low metal (A and D; n = 5), high vanadium (B and E; n = 5) and high lead (C and F; n = 5) zones of Nigeria. In the high vanadium and high lead zones, there was decreased density of immunoreactive neurons, and shrinkage of immunostained cell body and decreased proximal dendrites and neuropil, especially in high vanadium zone group. Also, there was significant loss (−41.8%) of SNc dopamine neurons in the animals exposed to high vanadium and (−50.7 %) and in those exposed to high lead, compared to those from low heavy metal zone (G). (***p < 0.001; ****p < 0.0001; NS not significant). Scale bar: 50 µm in A, B and C and 20 µm in D, E and F.

In the *substantia nigra pars compacta* (SNC) of AGR brains sampled from high vanadium (Fig. 1b, e) and high lead (Fig. 1c, f) concentration zones, the distribution of the populations of TH-immunoreactive neurons appeared reduced. Also, immunolabelling of dendrites and neuropil of AGR brains sampled from both high vanadium and high lead concentration zones appeared decreased compared to those sampled from low metals (Fig. 1a,d) concentration zone. Stereological cell counts of these TH cells showed a significant loss (−41.8 %) of SNC dopaminergic neurons in the animals exposed to high vanadium, and (−50.7 %) in those exposed to high lead, compared to those from low metals zone (Fig. 1g).

#### 3.2.2 Prefrontal (cingulate) cortex, hippocampus, dentate gyrus and reticular thalamic nuclei fast spiking GABAminergic interneurons (parvalbumin neurons)

**Fig. 2:**
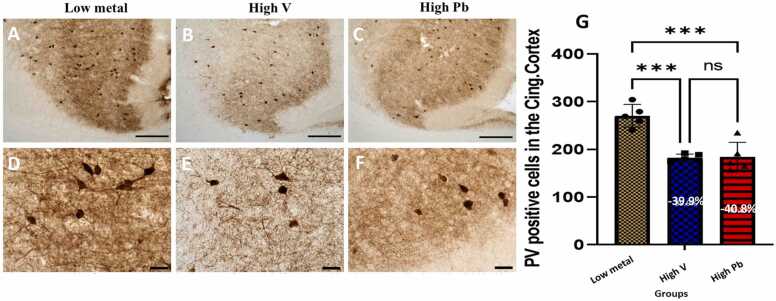
Immunoreactivity of fast-spiking inhibitory parvalbumin (PV) interneurons in cingulate cortex of AGR sampled from low metal (A and D; n = 5), high vanadium (B and E; n = 5) and high lead (C and F; n = 5) zones of Nigeria. In the high vanadium and high lead zones, there was decreased density of immunoreactive neurons, and shrinkage of immunostained cell body and decreased proximal dendrites and neuropil, especially in high vanadium zone group. Also, there was significant loss (−39.9 % and −40.8 %, respectively) of PV interneurons in the cingulate cortex of the animals exposed to high vanadium and those exposed to high lead, compared to those from low heavy metal zone (G). (***p < 0.001; NS not significant). Scale bar: 50 µm in A, B and C and 20 µm in D, E and F.

**Fig. 3:**
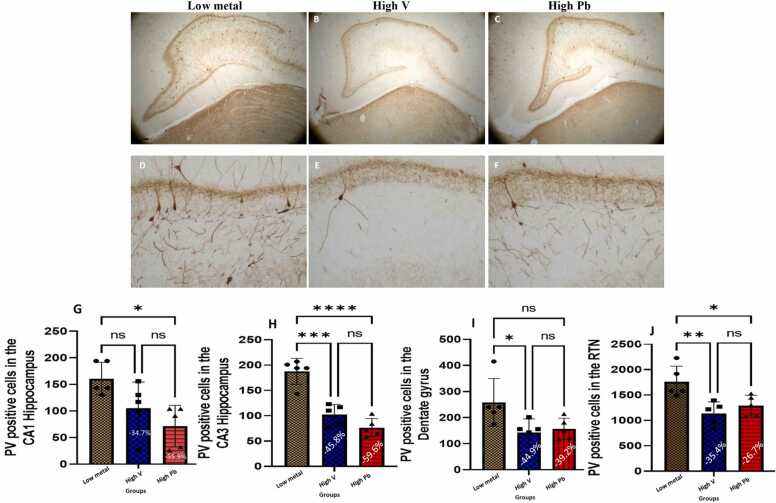
Immunoreactivity of fast-spiking inhibitory parvalbumin (PV) interneurons in hippocampus of AGR sampled from low metal (A and D; n = 5), high vanadium (B and E; n = 5) and high lead (C and F; n = 5) zones of Nigeria. In the high vanadium and high lead zones, there was decreased density of immunoreactive neurons, and shrinkage of immunostained cell body and decreased proximal dendrites and neuropil, especially in high lead zone group. Also, there was significant loss (−34.7 % and −55.9 %, respectively in the CA1 (G); −45.8 % and −59.6 %, respectively in the CA3 (H); −44.9 % and −39.2 %, respectively in the dentate gyrus (I); and −35.4 % and −26.7 %, respectively in the reticular thalamic nucleus (J)) of PV interneurons in the hippocampus and reticular thalamic neucleus of the animals exposed to high vanadium and those exposed to high lead, compared to those from low heavy metal zone. (*p < 0.05; **p < 0.01; ***p < 0.001; ****p < 0.0001; NS not significant). Scale bar: 50 µm in A, B and C and 20 µm in D, E and F.

The prefrontal cortex (Fig. 2), hippocampus (Fig. 3a-h), dentate gyrus (Fig. 2i) and reticular thalamic nuclei (Fig. 3j) of AGR brains sampled from high vanadium and high lead concentration zones, had reduced populations of parvalbumin-containing immunoreactive interneurons. Also, immunolabelling of dendrites and neuropil of AGR brains sampled from both high vanadium and high lead concentration zones appeared decreased compared to those sampled from low metals concentration zone. The soma of these parvalbumin-containing interneurons of AGR sampled from high vanadium and high lead concentration zones appeared swollen or thickened compared to those sampled from the low metal concentration zone. Stereological cell counts of these cells showed a significant loss (−39.9 %) of prefrontal cortex fast spiking GABAminergic interneurons in the animals exposed to high vanadium, and (−40.8 %) in those exposed to high lead, compared to those from low metals zone (Fig. 2g). For the different regions of the hippocampus and reticular thalamic nuclei, stereological count of PV cells showed a significant loss (−34.7% and −55.9% respectively) in the CA1 (Fig. 3g); (−45.8% and −59.6% respectively) in the CA3 (Fig. 3h); (−44.9 % and −39.2 % respectively) in the dentate gyrus (Fig. 3i) and (−35.4 % and −26.7 % respectively) in the reticular thalamic nuclei (Fig. 3j) of AGR exposed to high vanadium and those exposed to high lead respectively, compared to those from low metals region.

#### 3.2.3 Lateral hypothalamus (LH) orexinergic (OX-A) and Melanin concentration hormone (MCH) neurons

**Fig. 4:**
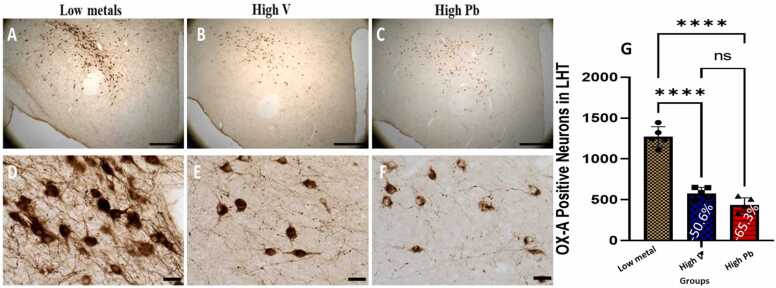
Immunoreactivity of Orexin-A neurons in the lateral hypothalamus of AGR sampled from low metal (A and D; n = 5), high vanadium (B and E; n = 5) and high lead (C and F; n = 5) zones of Nigeria. In the high vanadium and high lead zones, there was decreased density of immunoreactive neurons, and shrinkage and marked damage of immunostained cell body and decreased proximal dendrites and neuropil, especially in high lead zone group. Also, there was significant loss (−50.6% and −65.3%, respectively) of Orexin-A neurons in the lateral hypothalamus of the animals exposed to high vanadium and those exposed to high lead, compared to those from low heavy metal zone (G). (****p < 0.0001; NS not significant). Scale bar: 50 µm in A, B and C and 20 µm in D, E and F.

**Fig. 5:**
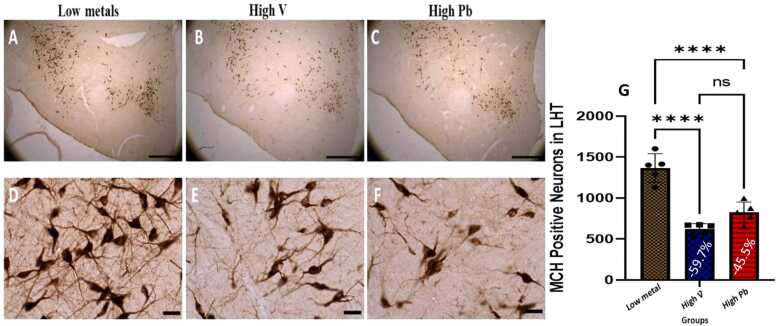
Immunoreactivity of melanin-concentrating hormone (MCH) neurons in the lateral hypothalamus of AGR sampled from low metal (A and D; n = 5), high vanadium (B and E; n = 5) and high lead (C and F; n = 5) zones of Nigeria. In the high vanadium and high lead zones, there was decreased density of immunoreactive neurons, and shrinkage and damage of immunostained cell body and decreased proximal dendrites and neuropil, especially in high lead zone group. Also, there was significant loss (−59.7 % and −45.5 %, respectively) of MCH neurons in the lateral hypothalamus of the animals exposed to high vanadium and those exposed to high lead, compared to those from low heavy metal zone (G). (****p < 0.0001; NS not significant). Scale bar: 50 µm in A, B and C and 20 µm in D, E and F.

In the lateral hypothalamus of AGR sampled from high vanadium and high lead concentration zones, the distribution of the populations of OX-A-immunoreactive (Fig. 4) and MCH-immunoreactive (Fig. 5) neurons was reduced and immunolabelling of dendrites and neuropil of AGR brains sampled from these zones appeared decreased compared to those sampled from low metals concentration zone similar to what was observed for TH and PV neurons. Stereological cell counts of OX-A neurons showed a significant loss (−50.6%) in the animals exposed to high vanadium, and (−65.3 %) in those exposed to high lead, compared to those from low metals zone (Fig. 4g). Similarly, a significant loss (−59.7 % and −45.5 % respectively) of MCH neurons in the lateral hypothalamus was observed in same animal groups compared to those of low metals region (Fig. 5g).

#### 3.2.4 Dendritic architecture of orexinergic (OX-A) neurons

**Fig. 6:**
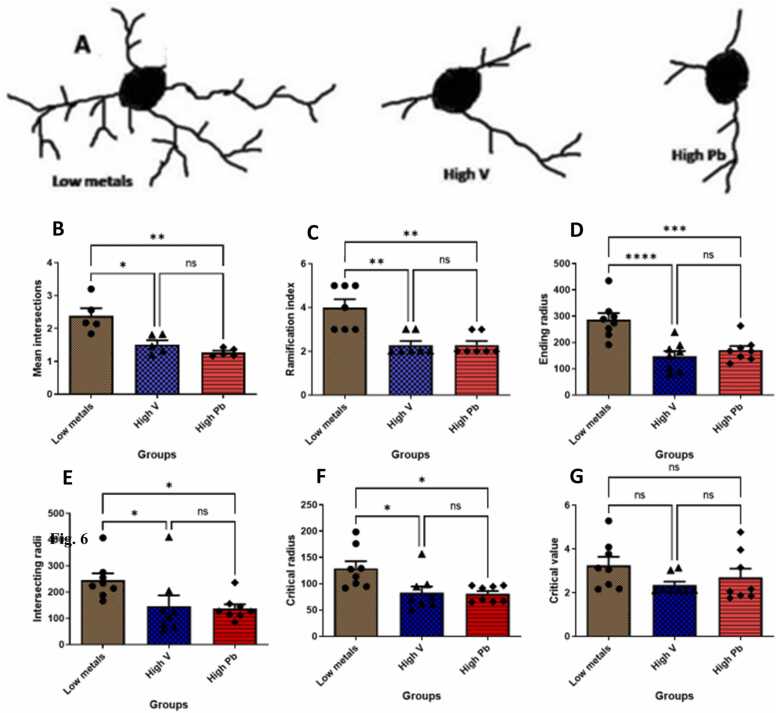
Quantitative analysis of the dendritic architecture of orexinergic neurons in AGR sampled from low metal, high vanadium and high lead zones of Nigeria. (A) Representative reconstructions of orexinergic neurons in AGR brains from low metals (n = 5), high vanadium (n = 5) and high lead (n = 5) zones of Nigeria. (B) Mean intersections, (C) Ramification index, (D) Ending radius, (E) Intersecting radii, (F) Critical radius, (G) Critical value as derived from Sholl analysis. (*p < 0.05; **p < 0.01; ***p < 0.001; NS not significant).

There was a significant decrease in the dendritic arborization of OX-A immunostained neurons in the animals exposed to high vanadium, and in those exposed to high lead, compared to those from low metals zone (Fig. 6a). Also, other parameters measured such as mean intersections (Fig. 6b), ramification index (Fig. 6c), ending, intersecting and critical radii (Fig. 6d-f respectively) were significantly decreased in the animals exposed to high vanadium, and in those exposed to high lead, compared to those from low metals zone. No significant difference was seen in the OX-A immunostained neurons dendritic critical value (Fig. 6g) of the animals exposed to high vanadium, and in those exposed to high lead, compared to those from low metals zone. This quantification therefore revealed a marked reduction of the complexity of dendritic arborization of OX-A-immunostained neurons in the animals exposed to high vanadium, and in those exposed to high lead in their natural habitat, compared to those from low metals zone.

#### 3.2.5 Perineuronal nets (PNNs) and extracellular matrix (ECM) alterations of fast spiking GABAminergic (Parvalbumin) interneurons

**Fig. 7:**
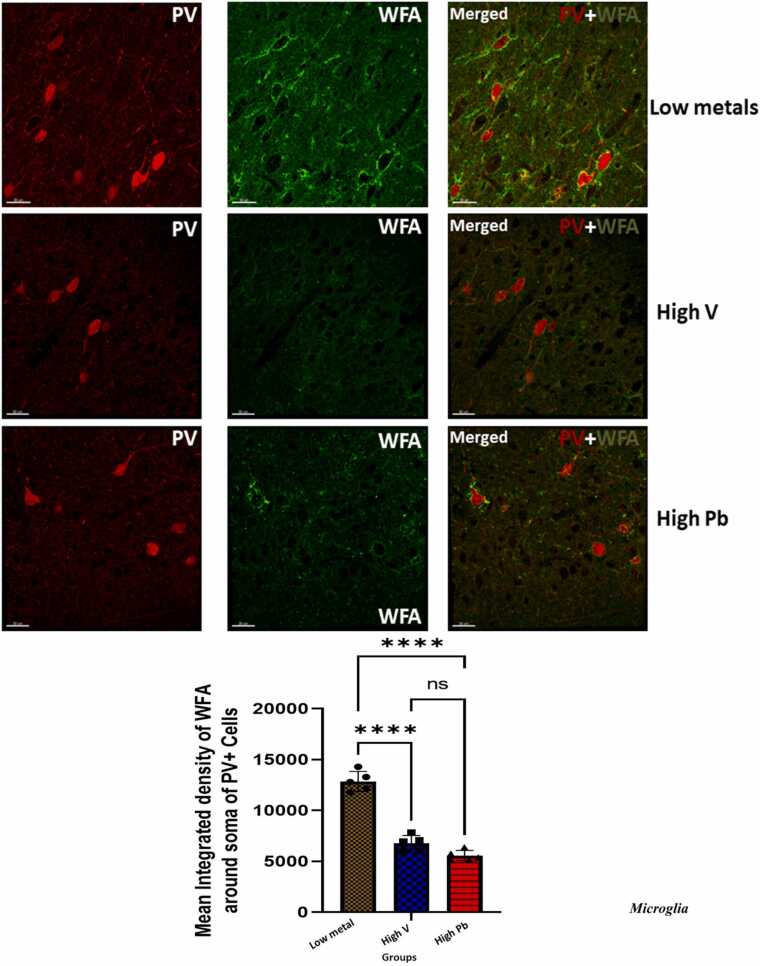
Densities of PV+, WFA+ and PV+WFA (merged) labeled cells in the prefrontal of AGR sampled from low metal (n = 5), high vanadium (n = 5) and high lead (n = 5) zones of Nigeria. A): Representative images of PV (in red), WFA (in green) and PV+WFA (merged)-positive cells in the prefrontal cortex. Upper panels show brain sections from low metals zone, middle panel show brain sections from high vanadium zone, while the lower panels depict sections from high lead zone. There was scanty and loss of PNN and ECM staining intensity in the high vanadium and high lead exposed zones compared to low metals zone. Also, there is significant decreased mean integrated density of WFA around soma of PV+ neurons in the high vanadium and high lead zones compared to low metal zone. (****p < 0.0001; NS not significant).

Our results showed a significant decreased integrated density of WFA around soma and dendrites of PV^+^ neurons in the animals exposed to high vanadium, and in those exposed to high lead, compared to those from low metals zone. Generally, there was scanty and loss of ECM staining intensity in same animal groups compared to those from low metals zone (Fig. 7).

#### 3.2.6 Microglia and astrocytes activation

**Fig. 8:**
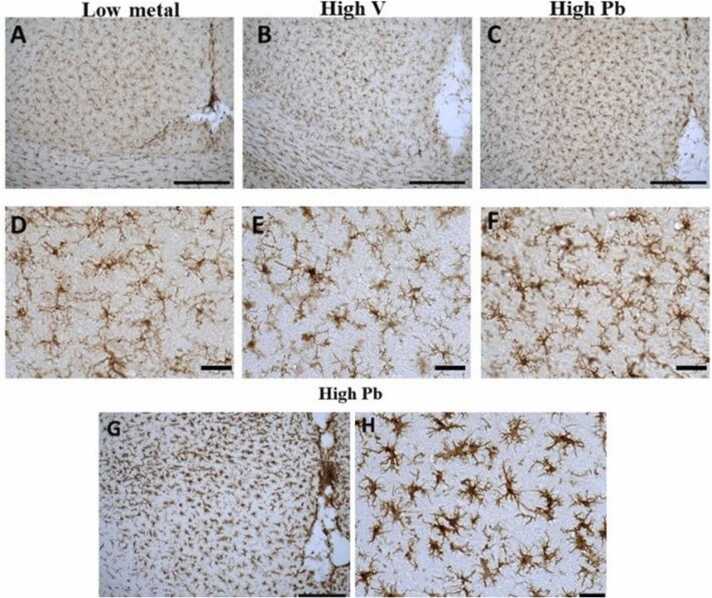
IBA-1 immuno-stained prefrontal cortex of AGR sampled from low metal (A and D; n = 5), high vanadium (B and E; n = 5) and high lead (C, F, G and H; n = 5) zones of Nigeria. Natural exposure to high vanadium and high lead resulted to relative microglial activation identified by an enlarged cell body with several short and thickened processes. G and H represent the so called “angry” microglia phenotype. Scale bar: 50 µm in A, B, C and G, and 20 µm in D, E, F and H.

AGR in high vanadium, and high lead groups had microglial cells showing increased Iba1 immunostaining; their cell body appeared hypertrophied, and their branches were hypertrophied, shorter and thicker, presenting a typical bushy appearance especially in the high lead group. These changes were seen in all regions of the brain (including the cortex, *substantia nigra*, lateral hypothalamus, corpus callosum and hippocampus) indicating their activation (Fig. 8) compared to those from low metals zone. In one of the brain samples from the high lead exposed AGR group, the microglia appeared amoeboid in shape, with their cell body extensively thickened, with thickened and retracted branches, presenting the so called “angry microglia” appearance (Fig. 8d).

**Fig. 9:**
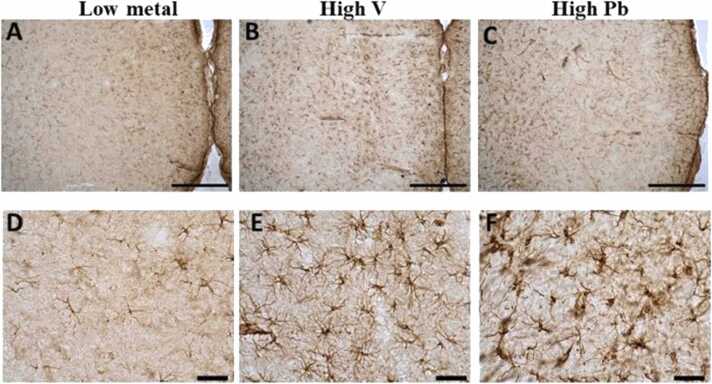
GFAP-immuno-stained prefrontal cortex of AGR sampled from low metal (A and D; n = 5), high vanadium (B and E; n = 5) and high lead (C and F; n = 5) zones of Nigeria. Natural exposure to high vanadium and high lead resulted to astrocytic activation identified by increased number of cells, thickened cell body with more extended cytoplasmic processes. Scale bar: 50 µm in A, B and C, and 20 µm in D, E and F.

Analyses of GFAP-positive astrocytes showed similar results to Iba 1. Astrocytes hypertrophy with enhanced GFAP immunostaining was observed in all regions of the brain in high vanadium, and high lead groups (including the cortex, *substantia nigra*, lateral hypothalamus, corpus callosum and hippocampus) indicating their activation compared to those from low metals zone (Fig. 9). The pathological changes were remarkably consistent in all brain samples of AGR from high vanadium exposed, and high lead exposed, compared to those from low metals zone. These microglia and astrocytic pathological changes were seen more in the high lead group.

### 3.3 Neurotoxicological findings in distinct neuronal cells of experimental vanadium exposed AGR, and in comparison with naturally exposed

#### 3.3.1 Substantia nigra pars compacta (SNC) and ventral tegmental area (VTA) dopaminergic neurons

**Fig. 10:**
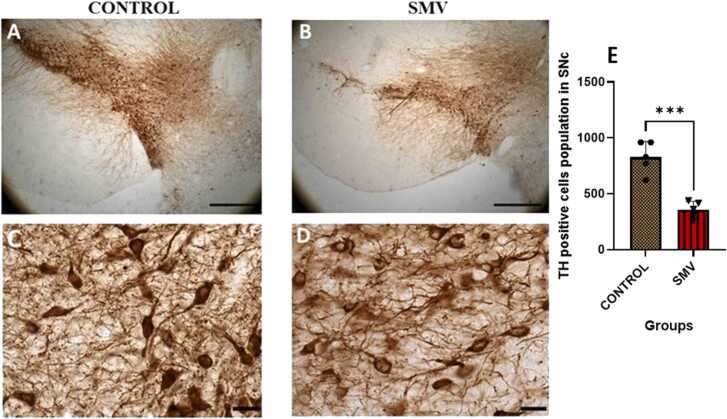
Tyrosine hydroxylase (TH) immunoreactivity of dopaminergic neurons in the substantia nigra pars compacta (SNc) of control AGR (A and C; n = 5) compared to 3 mg/kg body weight of intoxication with SMV for 14days (B and D; n = 5). In the SMV intoxicated group, there was decreased density of immunoreactive neurons, and shrinkage of immuno-stained cell body and decreased proximal dendrites and neuropil. Also, there was significant loss (−54.7 %) of SNc dopamine neurons in the SMV intoxicated group, compared to control (E). (***p < 0.001). Scale bar: 50 µm in A and B and 20 µm in C and D.

**Fig. 11:**
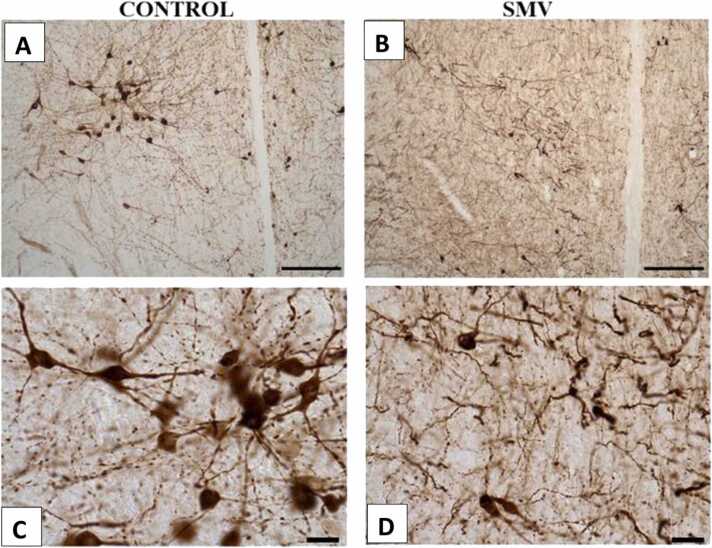
Tyrosine hydroxylase (TH) immunoreactivity of dopaminergic neurons in the ventral tegmental area (VTA) of control AGR (A and C; n = 5) compared to 3 mg/kg body weight of intoxication with SMV for 14days (B and D; n = 5). In the SMV intoxicated group, there was intense destruction of TH immunoreactive neurons, and shrinkage of immuno-stained cell body and decreased proximal dendrites and neuropil. Scale bar: 50 µm in A and B and 20 µm in C and D.

In the *substantia nigra pars compacta* (SNC) (Fig. 10) and ventral tegmental area (VTA) (Fig. 11) of AGR brains exposed experimentally to 3 mg/kg body weight SMV, the distribution of the populations of TH-immunoreactive neurons appeared reduced compared to control. Immunolabelling of dendrites and neuropil of these regions of the AGR brains treated with SMV appeared decreased compared to control (Fig. 10a-f) and stereological cell counts of these TH^+^ cells in the *substantia nigra pars compacta* (SNC) showed a significant loss (−54.7 %) of SNC dopaminergic neurons in the animals exposed to SMV compared to control (Fig. 10g).

#### 3.3.2 Prefrontal (cingulate) cortex, hippocampus, dentate gyrus and reticular thalamic nuclei fast spiking GABAminergic interneurons

**Fig. 12:**
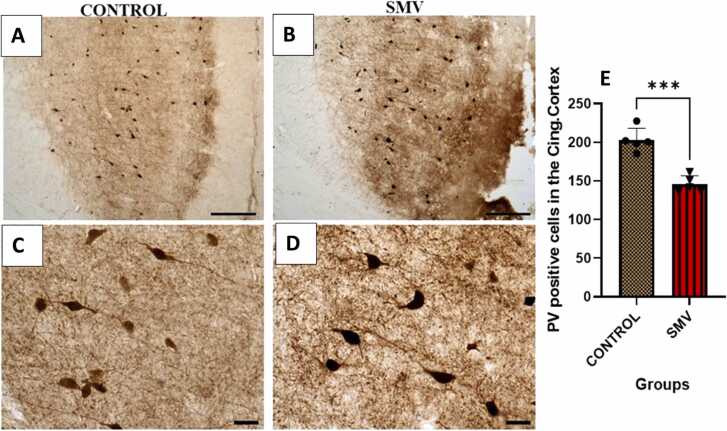
Immunoreactivity of fast-spiking inhibitory parvalbumin (PV) interneurons in cingulate cortex of control AGR (A and C; n = 5) compared to 3 mg/kg body weight of intoxication with SMV for 14days (B and D; n = 5). In the SMV intoxicated group, there was decreased density of immunoreactive neurons, and shrinkage of immune-stained cell body and decreased proximal dendrites and neuropil. Also, there was significant loss of PV interneurons in the cingulate cortex of the SMV intoxicated group, compared to control (E). (***p < 0.001). Scale bar: 50 µm in A and B and 20 µm in C and D.

**Fig. 13:**
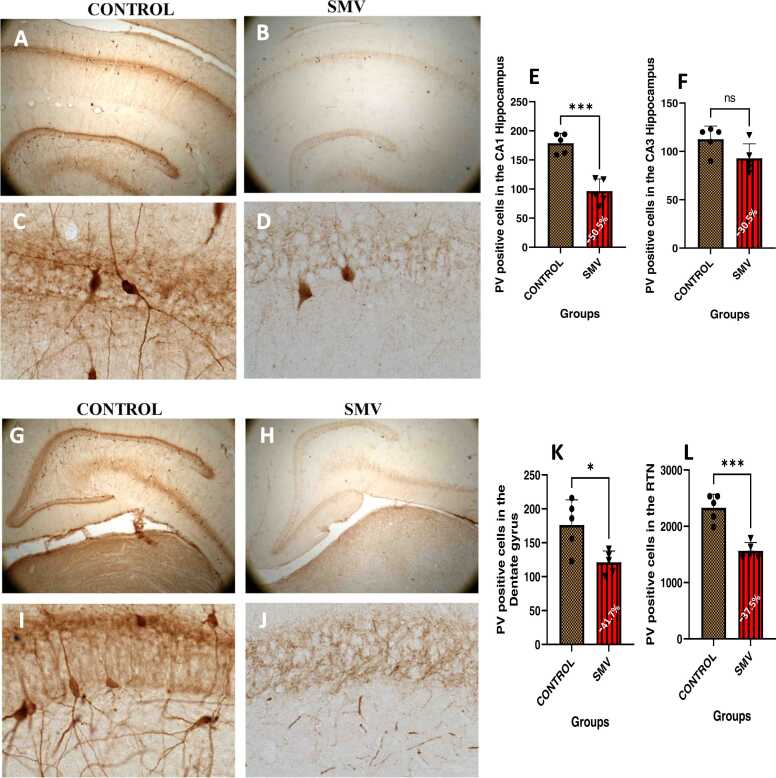
Immunoreactivity of fast-spiking inhibitory parvalbumin (PV) interneurons in the hippocampus and dentate gyrus of control AGR (A and C; and G and I respectively; n = 5) compared to 3 mg/kg body weight of intoxication with SMV for 14days (B and D; and H and J respectively; n = 5). There was a significant loss (−50.5% in the CA1 (E); − 41.7 % in the dentate gyrus (K) and 37.6 % in the reticular thalamic nucleus (L)) of PV interneurons in the SMV intoxicated group, compared to control. (*p < 0.05; ***p < 0.001; NS not significant). Scale bar: 50 µm in A, B, G and H and 20 µm in C, D, I and J.

In the prefrontal cortex (Fig. 12), hippocampus (Fig. 13a-h), dentate gyrus (Fig. 13i) and reticular thalamic nuclei (Fig. 13j) of AGR brains exposed experimentally to 3 mg/kg body weight of SMV, the distribution of the populations of parvalbumin-containing immunoreactive interneurons appeared reduced compared to the control match. Similar to what was observed for TH positive neurons, immunolabelling of dendrites and neuropil of AGR brains treated with SMV, they appeared decreased or destroyed compared to their control match. Stereological cell counts of these cells also showed a significant loss (−37.2 %) of prefrontal cortex fast spiking GABAminergic interneurons in the animals experimentally exposed 3 mg/kg SMV compared to control. A similar pattern was observed for different regions of the hippocampus (−50.5% in the CA1; −30.5 % in the CA3 and 41.7 % in the dentate gyrus) (Fig. 13g-i) and in the reticular thalamic nuclei (−37.6 %) (Fig. 13j), comparing the experimentally 3 mg/kg body weight SMV exposed group to their control match.

#### 3.3.3 Lateral hypothalamus (LH) orexinergic (OX-A) and Melanin concentration hormone (MCH) neurons

**Fig. 14:**
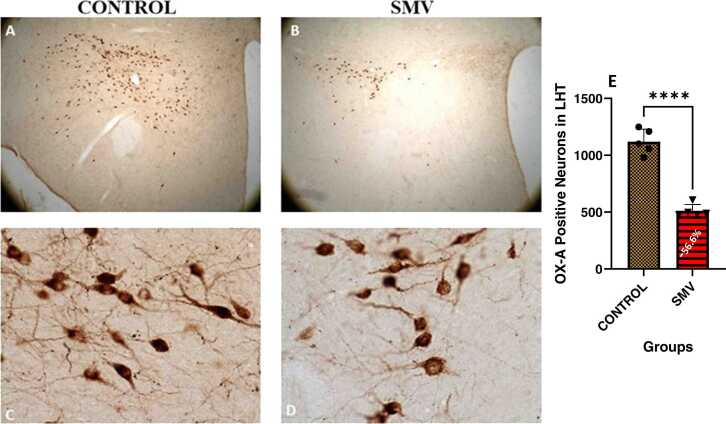
Immunoreactivity of Orexin-A neurons in the lateral hypothalamus of control AGR (A and C; n = 5) compared to 3 mg/kg body weight of intoxication with SMV for 14days (B and D; n = 5). In the SMV intoxicated group, there was decreased density of immunoreactive neurons, and marked damage of immuno-stained cell body and decreased proximal dendrites and neuropil compared to control. Also, there was significant loss (−56.6%) of Orexin-A neurons in the lateral hypothalamus of the AGR exposed to SMV compared to control (E). (****p < 0.0001). Scale bar: 50 µm in A and B and 20 µm in C and D.

**Fig. 15:**
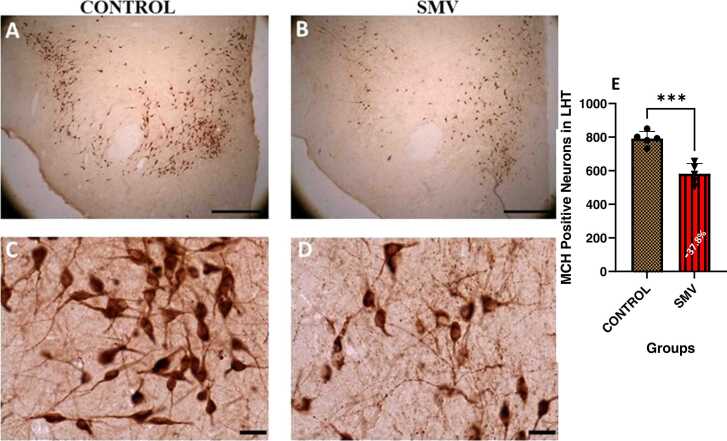
Immunoreactivity of melanin-concentrating hormone (MCH) neurons in the lateral hypothalamus of control AGR (A and C; n = 5) compared to 3 mg/kg body weight of intoxication with SMV for 14days (B and D; n = 5). In the SMV intoxicated group, there was intense destruction and decreased intensity of MCH immunoreactive neurons, and shrinkage of immuno-stained cell body and decreased proximal dendrites and neuropil compared to control. Also, there was significant loss (−37.8.7 %) of MCH neurons in the lateral hypothalamus of the animals exposed to SMV compared to control (E). (***p < 0.001). Scale bar: 50 µm in A and B, and 20 µm in C and D.

In the lateral hypothalamus of AGR brain exposed experimentally to 3 mg/kg body weight SMV, the distribution of the populations of OX-A-immunoreactive (Fig. 14) and MCH-immunoreactive (Fig. 15) neurons appeared reduced and immunolabelling of dendrites and neuropil of these neurons appeared decreased compared to control. Stereological cell counts of OX-A neurons showed a significant loss (−56.6 %) in the animals exposed experimentally to 3 mg/kg body SMV compared to control (Fig. 14e). Similarly, a significant loss (−37.8 %) of MCH neurons in same brain region in 3 mg/kg body SMV exposed group compared to control was observed (Fig. 15e).

#### 3.3.4 Dendritic architecture of orexinergic (OX-A) neurons

**Fig. 16:**
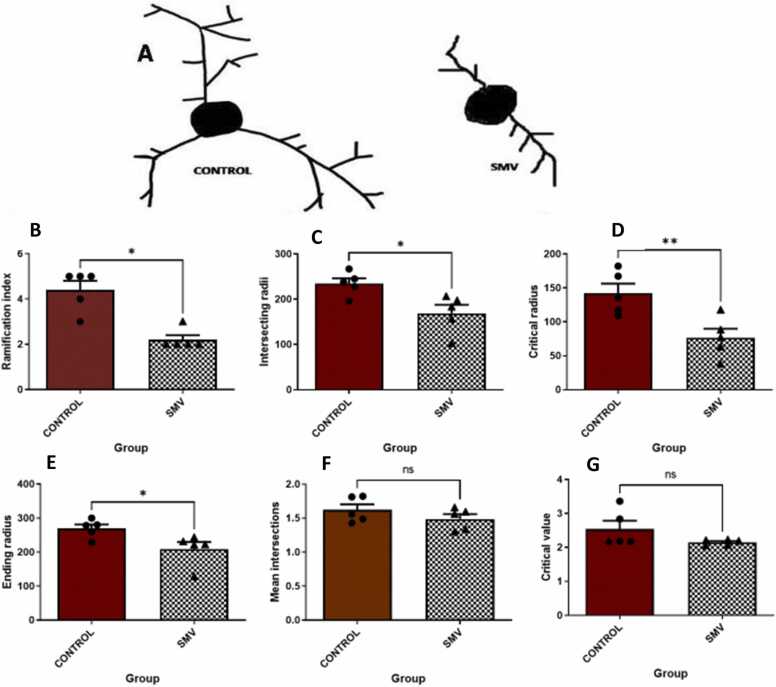
Quantitative analysis of the dendritic architecture of orexinergic neurons in AGR exposed to 3 mg/kg body weight SMV (n = 5) for 14days compared to control (n = 5). (A) Representative reconstructions of orexinergic neurons in control and 3 mg/kg body weight SMV exposed AGR. (B) Ramification index, (C) Intersecting radii, (D) Critical radius, (E) Ending radius, (F) Mean intersections (G) Critical value as derived from Sholl analysis. (*p < 0.01; **p ≤ 0.001; NS not significant).

The architecture of OX-A immunostained dendritic arbors showed a significant decrease in the AGR exposed experimentally to 3 mg/kg body weight SMV compared to control (Fig. 16a). A similar pattern (decreased) was noticed in ramification index (Fig. 16b), intersecting, critical and ending radii (Fig. 16c-e) in experimentally exposed group compare to control. However, no significant difference was noted in mean intersections and dendritic critical value in OX-A immunostained neurons of the experimentally 3 mg/kg body weight SMV exposed animals compared to control (Fig. 16f-g). This quantification revealed a marked reduction of the complexity of dendritic arborization of OX-A-immunostained neurons in the animals exposed experimentally to 3 mg/kg body weight SMV compared to control.

#### 3.3.5 Perineuronal nets (PNNs) and extracellular matrix (ECM) alterations of fast spiking GABAminergic interneurons

**Fig. 17:**
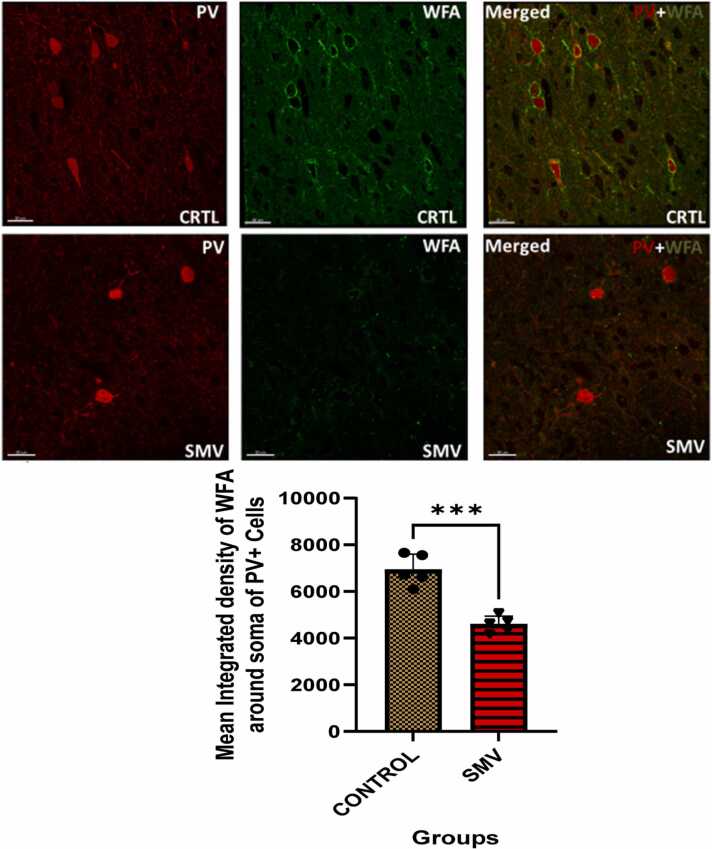
Densities of PV+, WFA+ and PV+WFA (merged) labeled cells in of AGR exposed to 3 mg/kg body weight SMV (n = 5) for 14days compared to control (n = 5). A): Representative images of PV (in red), WFA (in green) and PV+WFA (merged)-positive cells in the prefrontal cortex. Upper panels show brain sections from control, while the lower panels depict sections from 3 mg/kg body weight SMV exposed group. There was scanty and loss of PNN and ECM staining intensity in the SMV exposed group compared to control. Also, there was significant decreased mean integrated density of WFA around soma of PV+ neurons in the SMV exposed group compared to control. (***p ≤ 0.001; NS not significant).

Scanty and loss of ECM staining intensity was observed in SMV treated group compared to control and statistical analysis revealed a significant decreased mean integrated density of WFA around soma and dendrites of PV^+^ neurons in the animals exposed to 3 mg/kg body weight SMV compared to control match (Fig. 17).

#### 3.3.6 Microglia and astrocytes activation

**Fig. 18:**
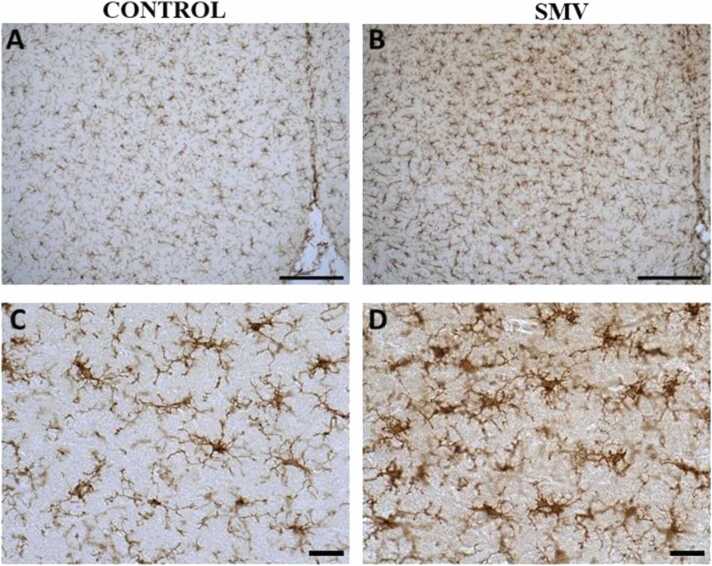
IBA-1 immuno-stained prefrontal cortex of AGR exposed to 3 mg/kg body weight SMV for 14days (B and D; n = 5) compared to control (A and C; n = 5). SMV intoxication resulted in microglial activation identified by an enlarged cell body with several short, thickened processes relative to the matched control. Scale bar: 50 µm in A and B, and 20 µm in C and D.

In the brains of AGR exposed experimentally to 3 mg/kg body weight SMV, microglial cells showing increased Iba1 immunostaining and cell body and their ramification hypertrophy were observed in almost all regions of the brain (including the cortex, *substantia nigra*, lateral hypothalamus, corpus callosum and hippocampus) studied indicating microglia activation compared to control match (Fig. 18).

**Fig. 19:**
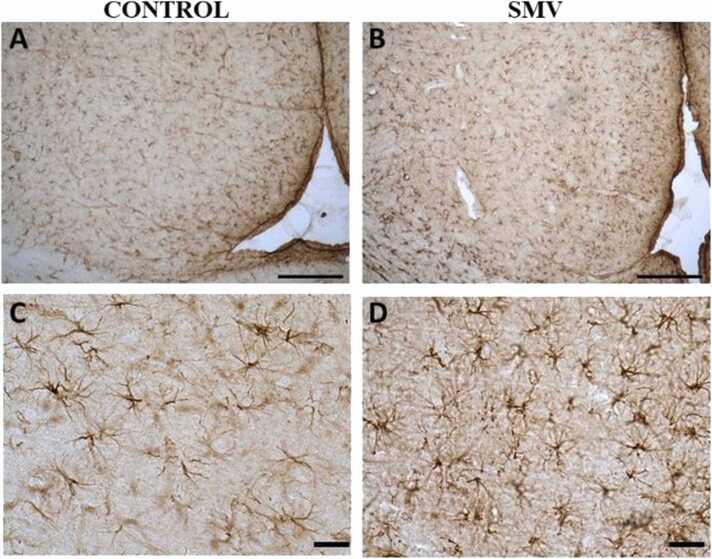
GFAP-immuno-stained prefrontal cortex of AGR exposed to 3 mg/kg body weight SMV for 14days (B and D; n = 5) compared to control (A and C; n = 5). SMV intoxication resulted in astrocytic activation identified by thickened cell body with more extended cytoplasmic processes relative to the matched control. Scale bar: 50 µm in A and B, and 20 µm in C and D.

Analyses of GFAP-positive astrocytes in brains of AGR exposed experimentally to 3 mg/kg body weight SMV, showed astrocytes hypertrophy with enhanced GFAP immunostaining in all regions of the brain (including the cortex, *substantia nigra*, lateral hypothalamus, corpus callosum and hippocampus) studied, indicative of astrocytes activation compared to control (Fig. 19). The pathological changes were remarkably consistent in all brain samples of experimentally SMV exposed AGR compare to control.

Statistical analysis showed no significant difference in all parameters measured comparing natural high vanadium zone and experimentally 3 mg/kg body weight SMV exposed groups.

### 3.4 Electron microscopic finding in brains of experimental 3 mg/kg body weight SMV exposed AGR

#### 3.4.1 Scanning electron microscopic findings

**Fig. 20:**
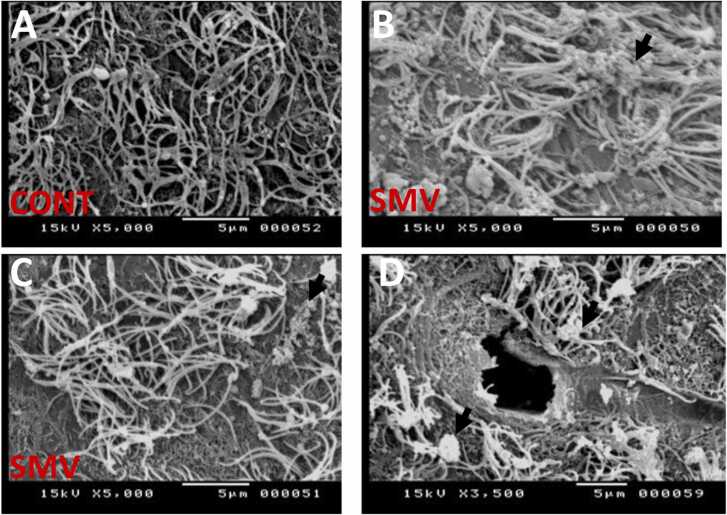
Scanning electron microscopy results for the floor of the lateral ventricle of control (A; n = 4) and 3 mg/kg body weight SMV intoxication of AGR for 14days (B, C and D; n = 4). Note the health cilia in the floor of the lateral ventricles of control AGR and the mass denudation and conglomeration (black arrow; B and C), general loss of cilia (B, C and D) and ruptured surface of the lateral ventricles in the SMV exposed group (blue arrow; D).

Scanning electron microscopic studies revealed severe pathological changes characterized by mass denudation, conglomeration and general loss of cilia on the surface of the lateral ventricles of brains of AGR exposed experimentally to SMV (Fig. 20b-d) compared to control that showed healthy and well spread cilia covering the surface of the lateral ventricle (Fig. 20a). The presence of ruptured surfaces (3/4) of the lateral ventricles was also noticed in brains of AGR exposed experimentally to SMV (Fig. 20d).

#### 3.4.2 Transmission electron microscopic findings

**Fig. 21:**
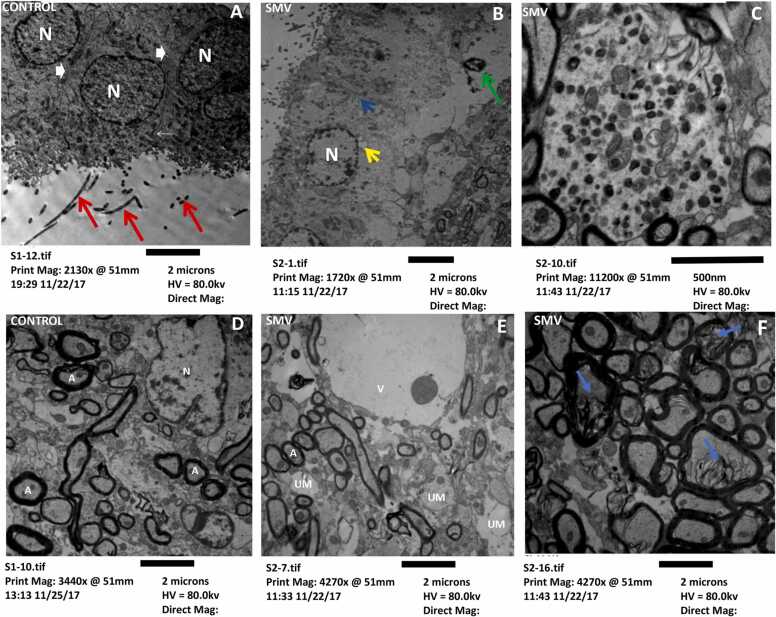
Transmission electron microscopy results for the subependymal layer of the lateral ventricle of control (a and d; n = 4) compared 3 mg/kg body weight SMV treatment of AGR for 14days (b, c, e and f; n = 4). b): a) Intact tight junction (white arrows), Ependymal cell nuclei (N), cilia (red arrow). b) Dissolution of tight junction between ependymal cells (blue arrow) (E), numerous vacuolations (yellow arrow). c): engulfed by activated microglia with large numbers of electron dense granules similar to granules of neutropil. d) Intact myelinated axons (A), Oligodendrocytes nucleus (N). e) demyelinated axon (UM) and dilated vessel (v). f) intense destruction of myelin sheath due to splitting of lamella of the sheath (blue arrow head).

Transmission electron microscopic studies also revealed severe pathological changes characterized by disintegration of the ependymal layer, dissolution of tight junction between ependymal cells and severe subependymal edema, intracytoplasmic membranous vesicle and numerous vacuolations (Fig. 21b) compared to control (Fig. 21a). These vesicles in some cases are very large, edematous and contains some protein materials (Fig. 21e) engulfed by activated microglia as large numbers of electron dense granules similar to granules of neutropil (Fig. 21c). Interesting finding in the experimental exposed SMV group is the intense destruction of myelin sheath due to splitting of lamella of the sheath (Fig. 21f) presenting numerous unmyelinated axons compared to control (Fig. 21 e).

## 4 Discussion

The present findings showed that AGR from their well-adapted but metals (vanadium and lead) polluted environment had significant loss of distinct neuronal cells sensitive to oxidative stress in different regions of the brain and damage of dendrites and extracellular matrix and perineuronal nests compared to AGR sampled from environment with low concentration of these metals. This current finding also suggests that vanadium treatment is a major contributor to these neuropathologies. The crucial lesions in these AGR brains with exposure to environmental heavy metals seem to be the neuronal damage, perhaps due to persistent chronic neuroinflammation. In a similar design but different focus, Calderon-Garciduenas et al. (2002) reported severe brain histopathologies in dog brains sampled from environmentally polluted South-west metropolitan Mexico City.

AGR from high vanadium and high lead concentration zones of Nigeria exhibit severe destruction of dopaminergic neurons of the *substantial nigra par compacta*, parvalbumin neurons in different brain regions, and orexinergic and MCH-containing neurons of the lateral hypothalamus presenting pictures that has been described in experimental animal models and humans exposed to different toxic substances and infectious agents like vanadium, lead, and *Trypanosoma brucei* etc (Kilduff and Peyron, 2000, Peyron et al., 2000, Lidsky and Schneider, 2003; Palomba et al., 2015; Guyon et al., 2009; Cabungcal et al., 2013; Ngwa et al., 2014).

Reduced distribution and destructions of dendrites and neuropil of the population of TH^+^ immunoreactive neurons in the SNc and VTA was a crucial finding in AGR sampled from those of high vanadium and high lead concentrated environments as well as AGR experimentally exposed to 3 mg/kg body weight SMV. Tyrosine hydroxylase is a marker for dopamine (a neurotransmitter) containing neurons (Weihe et al., 2006) and this present finding showed a down-regulation of dopamine biosynthesis in these AGR. This down-regulated dopamine observed could link to an array of abnormal neurobehavioural symptoms such as locomotor deficiency that may affect animals and humans living in these regions. Numerous experimental reports using heavy metals support this finding (Ngwa et al., 2014, Lidsky and Schneider, 2003). Specifically, vanadium is neurotoxic to doperminergic neurons in vitro and exposure to this metal in vivo have been shown to affect dopaminergic neurotransmitter system leading to depletion of dopamine (Ngwa et al., 2014, Deng et al., 2007, Atianjoh et al., 2008). Also, the neuropathologies of TH^+^ immunoreactive cells seen in the high lead zone could be due to the ability of this metal to disrupt the dopamine system leading to necrosis or apoptosis (Lidsky and Schneider, 2003). Exposure to high lead levels have been associated with variety of adverse effects especially on mechanism and structures of synapsis (Lidsky and Schneider, 2003, Jablonska et al., 1994), disrupting the activities of synatotagmin 1, a protein localized in the synaptic terminal and very important in the release of neurotransmitters (Bouton et al., 2001). Another effect of high lead exposure is its ability to alter neurotransmitter receptors eg glutamate receptor (Lidsky and Schneider, 2003) thus affecting hippocampal long term potentiation (Gilbert et al., 1996). The current findings of major destructions seen in the TH^+^ immunoractive cells in high vanadium and high lead zones suggest a potential risk of Parkinson’s disease characterized by progressive and substantive degeneration of dopaminergic neurons (Ngwa et al., 2014) among animals and humans living in these areas.

Parvalbumin neurons are fast spiking interneurons belonging to GABA cells subpopulation and controls basically output of principal neurons (Cabungcal et al., 2013). This PV group of neurons is necessary for fast rhythmic neuronal synchrony and facilitates information processing during cognitive task (Sohal et al., 2009, Whittington et al., 2011); (Cabungcal et al., 2013). The fast spiking nature of PV neurons imposes a high metabolic demand and increased mitochondrial density rending them sensitive to oxidative stress (Cabungcal et al., 2013). Several experimental reports have shown that vanadium and lead are associated with increased oxidative stress through generation of reactive oxygen species (Todorich et al., 2011, Azeez et al., 2016, Usende et al., 2016, Usende et al., 2018a, Usende et al., 2018b); Folarin et al., 2018), and thus the pathologies associated with PV immunopositive cells reported herein for AGR brains sampled from high vanadium and high lead concentration zones are similar to reports of Schiavone et al. (2009), Grillo et al. (2003) and Hu et al. (2010) on PV cells following severe environmental stress. In another study, the brains of AGR exposed experimentally to 3 mg/kg body weight SMV for 14 days showed increased oxidative stress and decreased levels of endogenous and exogenous antioxidants including GSH (Usende et al., 2018a, Usende et al., 2018b) similar to what was observed in the prefrontal cortex of human patients (Do et al., 2000, Wang et al., 2009, Gawryluk et al., 2011) with various mental conditions. The high metals level and environmental insults seen in the brains of AGR from these regions generated oxidative stress through the generation of reactive oxygen species (Folarin et al., 2018; Usende et al., 2018a, Usende et al., 2018b; Ejeh et al., 2019) which has contributed to the pathologies reported in the distinct neuronal cell types studied.

We also explore to know whether the intrinsic vulnerability of these metabolically intense PV neurons was due to the destruction of its very important unique protective mechanism of enwrappment with specialized extracellular matrix or aggrecan enriched perineuronal nets (PNNs). We reported herein the severe destruction of the ECM and PNNs around PV immunopositive cells in the high vanadium and high lead concentration zones and in the experimental exposed 3 mg/kg body weight SMV group. Perineuronal nets consist mainly of charged chondrotin sulfate proteoglycans hyaluronase tenascin and link proteins (Carulli et al. 2010) and are responsible for promotion of maturation of neurons as well as their synaptic and network stability (Sugiyama et al., 2009). PNNs are also known for their protection of neurons against oxidative stress (Morawski et al., 2004; Suttkus et al., 2012), a known pathway for vanadium and lead induced neuronal damage (Lidsky and Schneider 2003; Anderson et al., 1996; Todorich et al., 2011; Usende et al., 2016). Our findings especially in those exposed experimentally to 3 mg/kg body weight of SMV suggest that intact WFA labeled PNNs protects the fast spiking PV immunopositive cells against damage caused possibly due to oxidative stress (Usende et al., 2018a, Usende et al., 2018b). Concerning the findings of PNNs damage from brains of AGR sampled from high vanadium and high lead concentration zones, we cannot exclude the possibilities of other additional environmental stressors that might possibly contribute to the vulnerability of these neurons to damage. The present study and various other experimental studies (Morawski et al., 2004; Suttkus et al., 2012; Cabungcal et al., 2013) support the observations that PNNs are neuroprotective. Heavy metals including vanadium and lead cause brain damage through generation of ROS and increased lipid perioxidation (Lidsky and Schneider 2003; Anderson et al., 1996; Todorich et al., 2011). Due to their poly-anionic nature, PNNs are good chelators of iron (Morawski et al., 2004) which limits the formation of iron generated free radicals (Cabungcal et al., 2013). Also, the antioxidant properties of PNN due to its intact hyaluronan and chondroitin sulfate components (Campo et al., 2004, Canas et al., 2007) may be the reason for the neuronal protection seen in this study in the low metal zone and in the control group as these constituents of PNNs reduces the generation of hydroxyl radical (OH) through their ability to chelate transition metals limiting the initiation of both Fenton’s and Harber-Weiss reactions decreasing lipid perioxidation and neuronal damage (Cabungcal et al., 2013). Intact PNNs and ECM possibly serve to protect the neurons in the low metal and control groups by neutralizing ROS and boosting of their cellular antioxidant capacities.

In the present study, we showed a reduction of OX-A and MCH-containing immunopositive neuronal populations in AGR brains sampled from high vanadium and high lead zones and in AGR experimentally exposed to 3 mg/kg body weight SMV. These neuronal population reduction (together with that of TH and PV neuronal) may be attributed to either cell death or increase peptide release or down regulation of peptide expression as documented by Palomba et al. (2015) in their experimentally induced sleeping sickness model. We demonstrate striking decreased number of dendritic arborization and other structural damages of OX-A neuronal population of AGR brains sampled from high vanadium and high lead zones and well as in SMV intoxicated model. This finding favours the occurrence of neurodegenerative process of neurons of AGR sampled from these high metals zones and following SMV intoxication. Similar neuronal structural damages have been reported of OX-A neurons in mice experimentally induced with sleeping sickness by infection with *Trypanosoma brucie* parasites (Polomba et al., 2015). Neurodegenerative disorders and other conditions such as mental retardation, alcoholism and epilepsy have been associated with decreased neuronal arborization and spine (Fiala et al., 2002) as documented herein with OX-A neurons. Previously, Avila-Costa et al. (1999) reported neuronal spine loss in the hippocampus after exposure to ozone and also following vanadium inhalation (Avila-Costa et al., 2004, Avila-Costa et al., 2006) similar to our present sholl analysis result after intraperitoneal exposure protocol. The current finding concerning the OX-A neurons and together with the MCH-containing neurons, TH^+^ and PV^+^ neuronal populations reduction suggests the potential vulnerability of these cells to chronic inflammation.

Microglia are mediators of neuroinflammatory processes (Hanisch and Kettenmann, 2007; Azeez et al., 2016) and the present study showed microglia activation in brains of AGR sampled from high vanadium and high lead zones as well as SMV intoxicated group with a distribution matching that of astrocytes, confirming neuroinflammation. The microglia activation reported herein also correlate well with the myelin and axonal damage seen in our ultrastructural studies. Interestingly, one brain sample from high lead zone showed an “angry” amoeboid microglia phenotype indicating that the inflammation has occurred over a long period of time (Hanisch and Kettenmann, 2007). Studies have shown that activation of microglia is neurotoxic (Jha et al., 2016) and that microglia recruitment is regulated by astrocytes (Skripuletz et al., 2013, Gudi et al., 2014), therefore, the need for astrocytes activation reported herein.

Astrocytes are known for their role in regulation of neuronal (including synaptic) microenvironment and in defending the brain against toxic and oxidative insults (Sofroniew and Vinters, 2010, Heller and Rusakov, 2015) therefore, their activation in AGR brains sampled from high vanadium and high lead as well as in experimentally intoxicated SMV group is suggestive of oxidative insults and this correlate well with the loss of neuronal populations sensitive to oxidative stress. Astrogliosis have previously been reported in vanadium neurotoxicity (Garcia et al., 2005, Todorich et al., 2011, Mustapha et al., 2014, Azeez et al., 2016, Usende et al., 2016). Although astrocytes together with microglia are critical in the process of iron homeostasis crucial for the synthesis of myelin (Clemente et al., 2013, Lundgaard et al., 2014), astrocytes are known for their inhibitory effects on remyelination (Alizadeh et al., 2015).

As reported by Avila-Costa et al. (2005), we show using electron microscopy that vanadium enters the brain and induces structural damages of the ependymal layer characterised by severe loss of cilia, mass denudations, sloughing of cells and detachment of the ependymal layer. Interestingly, we showed disruption of the ependymal cells junctions as a pathological pathway and rupture of the surface of the lateral ventricles for the first time following SMV intoxication. This rupture is currently unique to the AGR model of vanadium neurotoxicity. The cause of this rupture however, remains to be investigated. The intense destructions of myelin sheath and neuronal axons seen confirm earlier report of Azeez et al. (2016) of myelin and axonal damage using SMI-32 biomarker after vanadium intoxication in mice model. Hypomyelination as reported here is a major pathological presentation of vanadium intoxication (Soazo and Garcia, 2007, Todorich et al., 2011, Mustapha et al., 2014, Azeez et al., 2016, Usende et al., 2016) due to the vulnerability of the oligodendrocyte progenitor cells to iron homeostasis and assimilation, a key event disrupted following vanadium toxicity resulting from generation of reactive oxygen species and apoptosis (Todorich et al., 2011, Usende et al., 2016). It is interesting to note that the myelin damage reported herein is at the subependymal region of the brain. Myelin damage has been previously shown to occur in the midline of the corpus callosum, and in the gray region of the diencephalon, hippocampus and neocortex after vanadium intoxication (Azeez et al., 2016) and in cuprizone model of demyelination (Clarner et al., 2012).

## 5 Conclusion

In conclusion, the present study has revealed hallmarks of destructions of distinct neuronal cells sensitive to oxidative stress and neuroinflammation in response to natural environmental exposure to heavy metals (vanadium and lead) and based on results of experimental vanadium intoxication, this study showed the contribution of vanadium in this damage. Altogether, the study has provided relevant data in the new field of neuroecotoxicology, of utmost importance for brain health in polluted regions. These to our knowledge are the first “neuroecotoxicological” findings in distinct neuronal cell groups in the AGR. The implications of these findings are highly relevant for the human population living in these areas, not only in Nigeria but also in similarly polluted areas elsewhere in the world.

## Ethics approval

All animal experiments were carried out under ethical approval of the University of Ibadan Animal Care and Use Research Ethics Committee Review (UI-ACUREC/18/0059) and in accordance with ethical standard of the National Institute of Health Guide for the Care and Use of Laboratory Animals (NIH Publications No. 80–23) revised 1996 and the European Communities Council Directive of November 24, 1986 (86/609/EEC).

## Funding

This work was partly supported by the University of Verona Internationalisation Programme; Mobility and Development Cooperation to Dr. Usende Ifukibot Levi and Prof Marina Bentivoglio; and the Institution Based Research (IBR), Tertiary Education Trust Fund (TETFund) of University of Abuja, Reference No: TETFUND/DESS/UNI/ABUJA/RP/VOL 1 to Dr. Usende Ifukibot Levi.

## CRediT authorship contribution statement

**Conceptualization**: James Olukayode Olopade, Ifukibot Levi Usende, Marina Bentivoglio; **Methodology:** Ifukibot Levi Usende, Idris Ayodeji Azeez, Anna Andrioli; **Formal analysis and investigation**: Ifukibot Levi Usende, Molakun Bankole; **Writing** - original draft preparation: Ifukibot Levi Usende; **Writing - review and editing:** Ifukibot Levi Usende, James Olukayode Olopade, Funmi Olopade; **Funding acquisition:** Ifukibot Levi Usende, Marina Bentivoglio; **Supervision:** James Olukayode Olopade.

## Conflicts of Interest

The authors declare no conflict of interest.
